# The Role of Autophagy and Related MicroRNAs in Inflammatory Bowel Disease

**DOI:** 10.1155/2018/7565076

**Published:** 2018-06-04

**Authors:** Shiyuan Wang, Yan Huang, Cili Zhou, Huangan Wu, Jimeng Zhao, Luyi Wu, Min Zhao, Fang Zhang, Huirong Liu

**Affiliations:** ^1^Key Laboratory of Acupuncture-Moxibustion and Immunology, Shanghai University of Traditional Chinese Medicine, Shanghai 201203, China; ^2^Shanghai Research Institute of Acupuncture and Meridian, Shanghai 200030, China

## Abstract

Accumulating evidence demonstrates that microRNA- (miR-) mediated posttranscriptional regulation plays an important role in autophagy in inflammatory bowel disease (IBD), a disease that is difficult to manage clinically because of the associated chronic recurrent nonspecific inflammation. Research indicates that microRNAs regulate autophagy via different pathways, playing an important role in the IBD process and providing a new perspective for IBD research. Related studies have shown that miR-142-3p, miR-320, miR-192, and miR-122 target *NOD2*, an IBD-relevant autophagy gene, to modulate autophagy in IBD. miR-142-3p, miR-93, miR-106B, miR-30C, miR-130a, miR-346, and miR-20a regulate autophagy by targeting *ATG16L1* through several different pathways. miR-196 can downregulate *IRGM* and suppress autophagy by inhibiting the accumulation of LC3II. During the endoplasmic reticulum stress response, miR-665, miR-375, and miR-150 modulate autophagy by regulating the unfolded protein response, which may play an important role in IBD intestinal fibrosis. Regarding autophagy-related pathways, miR-146b, miR-221-5p, miR-132, miR-223, miR-155, and miR-21 regulate NF-*κ*B or mTOR signaling to induce or inhibit autophagy in intestinal cells by releasing anti- or proinflammatory factors, respectively.

## 1. Introduction

Inflammatory bowel disease (IBD) is divided into Crohn's disease (CD) and ulcerative colitis (UC). As a chronic nonspecific disease, the recurrent and persistent features of IBD are difficult to comprehensively cure, and the disease has a serious impact on patient quality of life. With the acceleration of urbanization and modernization, the incidence of IBD has increased to more than 0.5% of the total population of developed countries, such as in Europe and the United States [1]. The prevalence of IBD has also risen significantly in Asia, with rates in East Asia more than doubling in the past few decades [2]. IBD is generally accepted to be caused by interactions among immune deficiencies, genetic factors, and environmental factors in susceptible populations, though the specific pathogenesis remains unclear [3]. Based on recent molecular studies, researchers are recognizing that genetic factors involved in the inflammatory response and immune function and related pathways (including microRNAs and autophagy) play important roles in the pathophysiology of IBD [4].

MicroRNAs (miRNAs) are evolutionarily conserved, endogenous, single-stranded, noncoding RNAs that bind to the 3′ untranslated region (UTR) and 5′ UTR or partially translated region of a target mRNA [5], inhibiting transcription of the mRNA by blocking its translation [6]. Wu et al. [7] first evaluated abnormal expression of miRNAs in the intestinal tissue of UC patients in 2008, finding a specific miRNA expression pattern: three miRNAs (miR­192, miR­375, and miR­422b) were markedly downregulated and eight (miR­16, miR­21, miR­23a, miR­24, miR­29a, miR­126, miR­195, and let­7f) notably upregulated in active UC tissues [8]. Since then, further studies have confirmed that miRNAs participate in the occurrence and development of IBD (including inactive UC) through the immune system, related inflammatory pathways, and other pathways. In addition to being related to colon cancer and inflammatory-associated cell senescence, miR-21 expression is significantly increased in intestinal fibroblasts and may participate in the IBD process through NOS2 and CD68 [9].

Autophagy is a eukaryotic cellular response to starvation, hypoxia, toxicity, other external pressures, or other stimuli. The process involves the formation of a membrane surrounding internal structures, such as organelles and cytoplasmic macromolecules, phagocytosis of intracellular components, and their presentation to the lysosome for degradation and recycling. Although the main function of autophagy is to maintain cellular homeostasis [10, 11], it also plays an important role in host defense, especially in regulating inflammation [12, 13]. Autophagy defects can lead to a series of problems, including intestinal epithelial dysfunction, innate immune dysfunction, and intracellular pathogen clearance. For example, when autophagy is defective, adherent-invasive *Escherichia coli* (AIEC) is not addressed by the cell, and proinflammatory cytokine secretion (such as TNF-*α* and IL-6) increases in the intestinal tract [14, 15].

**Figure 1:**
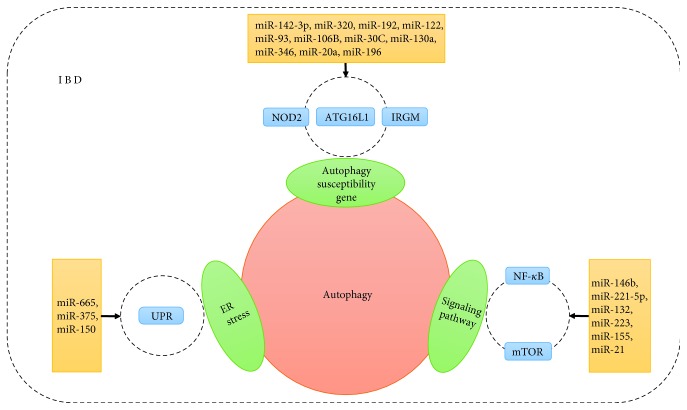
miRNAs regulate cell autophagy via different molecular pathways in the process of IBD.

There is also recent evidence showing that miRNAs regulate autophagy via different molecular pathways and have key roles in IBD [16–20]. miRNAs can regulate intestinal autophagy by targeting IBD-relevant autophagy genes such as *NOD2*, *ATG16L1*, and *IRGM*, thus modulating innate intestinal immunity and intestinal epithelial function. These miRNAs are also involved in autophagy by regulating the unfolded protein response (UPR) during endoplasmic reticulum stress, which contributes to IBD intestinal fibrosis. Studies of cellular pathways have found that miRNAs can induce or inhibit intestinal autophagy by regulating NF-*κ*B and mTOR signaling, thereby affecting inflammatory factors and anti-inflammatory or proinflammatory effects (Table 1 and Figure 1).

Several review articles to date have discussed the role of miRNAs in IBD [8, 16, 18, 19, 21–25], However, there are no reviews that focus on the IBD field and on miRNAs that regulate autophagy and associated pathways. Elucidation of the interaction between miRNAs and autophagy in IBD-specific mechanisms will help to explain the occurrence, development, and future molecular targets of IBD therapy. This article aims at summarizing the mechanism of miRNAs related to IBD that regulate autophagy via different pathways and to provide a theoretical reference for further research.

## 2. IBD-Relevant Autophagy Genes

Widely accepted IBD-relevant autophagy genes, including *NOD2*, *ATG16L1*, and *IRGM*, were identified through genome-wide association studies (GWASs) and subsequent meta-analyses of GWAS and immune chip data [26]. These studies have been crucial to elucidating the mechanisms underlying IBD, particularly autophagy and innate immunity in CD and intestinal epithelial barrier dysfunction in UC. They have also provided clues for novel IBD therapeutic strategies [18].

### 2.1. *NOD2*

*NOD2* is the first gene thought to predict increased susceptibility to CD. The *NOD2* protein is a cytoplasmic receptor that senses bacterial wall peptides and promotes clearance by initiating proinflammatory transcription [27]. Because *NOD2* signaling can activate autophagy, it has recently been shown that the *NOD2* pathway and autophagy are cross-regulated, and this relationship plays a positive role in intracellular bacterial clearance [28, 29].

It has also been reported that miR-142-3p effectively inhibits autophagy in a NOD2-dependent manner and downregulates IL-8 mRNA expression [30]. There is a negative correlation between *NOD2* and miR-320 expression: miR-320 downregulates *NOD2* mRNA and protein expression. In IBD, expression of miR-320 in intestinal mucosa is significantly decreased, which may explain the observed rise in *NOD2* expression [31, 32]. Researchers have also identified the interaction between miR-192 and *NOD2* as possibly being related to the pathogenesis of IBD. miR-192, the most highly expressed UC-associated miRNA, appears to be dysfunctional in the intestinal epithelial cells of patients with IBD. Additionally, miR-192 significantly alters expression of *NOD2* mRNA and protein and significantly reduces phosphorylation of NF-*κ*B and expression of IL-8 and CXCL3 [17, 33].

As NF-*κ*B has an inhibitory effect on autophagy (see Section 3.1) [34], it may have a key function in the pathogenesis of IBD and the innate immune response of intestinal epithelial cells [33]. miR-122 regulates the PI3K/Akt/mTOR/p70S6K pathway in breast cancer and downregulates *NOD2* expression [35]. In CD, miR-122 targets *NOD2* and reduces lipopolysaccharide- (LPS-) induced apoptosis by inhibiting *NOD2*, a process that activates the NF-*κ*B pathway. Because activation of NF-*κ*B has an inhibitory effect on autophagy, miR-122 can play an autophagy-related role in IBD [36]. In addition, by regulating *NOD2* and proinflammatory cytokines, miR-146a may be important in IBD; however, it still needs to be verified whether miR-146a affects autophagy via *NOD2* [37].

### 2.2. *ATG16L1*

*ATG16L1* is an important adapter protein in the autophagosome formation that occurs during autophagy [38]. Some human and animal studies have shown that *ATG16L1* dysfunction is closely related to intestinal inflammation in CD [16, 39]. miR-142-3p was recently shown to negatively regulate *ATG16L1* in CD colon epithelial cells. Upregulation of miR-142-3p decreases expression of *ATG16L1* mRNA and protein, thereby reducing the autophagic activity of related cells [30, 40]. AIEC infection can lead to overexpression of miR-93 and miR-106B, inhibition of *ATG16L1*, and downregulation of *ATG5*, causing a reduction in autophagosome formation and thus interfering with the autophagy pathway and bacterial clearance [41]. In intestinal epithelial HCT116 cells, miR-106b targets *ATG16L1* and modulates autophagy, partially through a binding site at the 3′ end of the *ATG16L1* 3′ UTR. In addition, miR-106a-regulated autophagy may also occur in an *ATG16L1*-independent manner [42]. In ovarian cancer cells, NF-*κ*B transcriptionally upregulates miR-130a expression in response to inflammatory stimuli, and miR-130a can increase levels of p-mTOR and impair LC3-II accumulation, leading to blockade of rapamycin- or starvation-induced autophagy [43]. AIEC also enhances expression of miR-30C and miR-130a by activating NF-*κ*B, resulting in the downregulation of *ATG5* and *ATG16L1* and inhibition of autophagy. This study also revealed that the signaling pathway through which this occurs may be effective in inducing the proinflammatory cytokine IL-8, leading to defects in autophagy-mediated clearance of AIEC [44].

Vitamin D receptor (VDR) activation downregulates expression of *ATG16L1* and its related lysozyme, impairing the antibacterial effect of Paneth cells and resulting in defective autophagic function in intestinal inflammatory cells [45]. VDR is the target of miR-346, and TNF-*α* induces miR-346 expression during intestinal mucosal inflammation, which downregulates VDR in the intestinal epithelium and affects autophagy [46]. Gene set enrichment analysis (GSEA) has demonstrated that miR-20a expression is negatively correlated with the autophagy-lysosome pathway. Indeed, miR-20a regulates several genes related to autophagy and inhibits *ATG16L1*, *BECN1*, and *SQSTM1* protein expression [47]. One study found miR-20a to be significantly increased in the intestinal mucosa of pediatric CD patients [48].

### 2.3. IRGM

Immunity-related GTPase family M protein (IRGM) has been considered to be associated to autophagy since 2006, though its specific molecular association with autophagy remains unclear [49]. More recent studies have shown that *IRGM* proteins play important roles in innate immunity against intracellular pathogens (such as CD-associated AIECs) [15, 50]. *IRGM*-dependent autophagy plays an important role in combating pathogenic AIEC, and the abundance of pathogenic AIEC has been demonstrated to be higher in the intestinal mucosa of CD patients than in that of healthy controls [51]. Furthermore, studies have shown that *IRGM* regulates autophagy by affecting mitochondrial division [52]. By regulating *IRGM* expression, miR-196 may participate in the endogenous fine-tuning of autophagic pathway initiation and in the control of intracellular pathogen degradation in human cells. miR-196 has also been shown to be overexpressed in inflammatory intestinal epithelial cells in CD. This expression downregulates the *IRGM* protective variant (c.313C) but not the risk-associated allele (c.313T), suggesting that CD-associated risk (T allele) and protective (C allele) haplotypes confer differences in *IRGM* expression under the control of miR-196. Notably, this regulatory mechanism does not appear to occur in all cell types. Additionally, overexpression of miR-196 can also induce downregulation of LC3II transformation, inhibiting LC3II accumulation. In other words, miR-196 inhibits autophagy from its initiation step. Downregulation or loss of *IRGM* expression may also impair the control of autophagy (xenophagy) of CD-associated AIEC in cellular replication [53].

## 3. Endoplasmic Reticulum Stress Pathway

### 3.1. Effect of Endoplasmic Reticulum Stress on Autophagy in IBD

**Figure 2:**
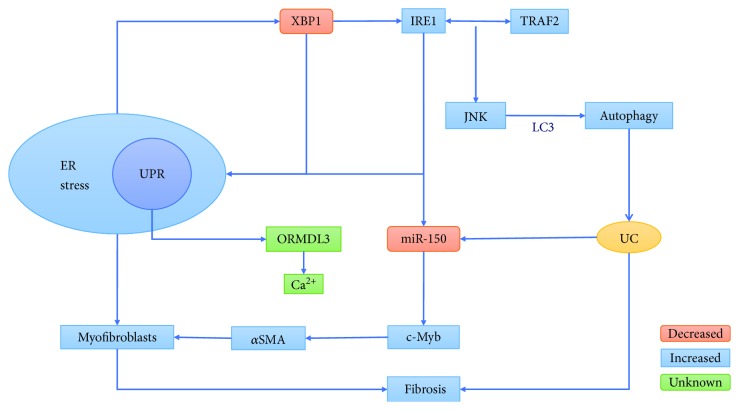
Endoplasmic reticulum stress and autophagy.

GWASs have identified endoplasmic reticulum (ER) stress as being associated with IBD because such stress is increased in inflammatory intestinal epithelial cells [54]. ER stress is a cellular process that is triggered by the folding of multiple interfering proteins in the endoplasmic reticulum. This process allows cells to cope with an excess of misfolded or expanded proteins through a complex signaling network known as the UPR [55], which can rebalance the ER through an adaptive mechanism involving autophagy. Defects in autophagy invoke the ER stress response [56], promoting autophagy [57]. *XBP1* and *ORMDL3* are two genes involved in UPR that are thought to be associated with IBD, and lack of their expression can promote intestinal inflammation [58, 59]. Deletion of *XBP1* enhances activation of IRE1 and sensitizes intestinal epithelial cells to inflammatory cytokines [59]. Directly upstream of *XBP1*, IRE1 is a kinase that can induce activation of JNK signals under ER stress through interaction with TRAF2 [60]. JNK directly induces formation of autophagosomes by LC3 (Figure 2) [61]. *ORMDL3* regulates Ca^2+^ uptake in the ER, and *ORMDL3* dysfunction results in protein misfolding and increased sensitivity to ER stress. The underlying mechanism of these molecular interactions with regard to intestinal inflammation remains unclear [62, 63].

### 3.2. Regulation of miRNAs via the ER Stress Pathway

Studies have shown that miRNAs can regulate ER stress and induce autophagy [64]. miR-665 has been shown to be upregulated in the intestinal mucosa of patients with IBD and can downregulate expression of *XBP1* and *ORMDL3* during ER stress, increasing JNK activity and leading to an increase in inflammatory factor-induced apoptosis and autophagy sensitivity. Additionally, miR-665 may be important in the positive feedback regulation of the miR-665/ER/NF-*κ*B loop, resulting in chronic inflammation of the gastrointestinal tract, but the mechanism requires further validation [65]. Expression of miR-375, which regulates ER stress, is elevated in normal colon tissue and significantly reduced in intestinal inflammation [66, 67]. However, because its specific mechanism is not clear, involvement of miR-375 in regulating autophagy in IBD through ER stress modulation requires further study. In HeLa cells, miR-346 was found to be induced under ER stress and to modulate autophagic flux. GSK3B has been shown to be the target of miR-346 and to participate in ER stress-related autophagy. miR-346 activates autophagy by interrupting the association between *BCL2* and *BECN1* in a GSK3B-dependent manner. However, whether this mechanism plays a role in IBD remains to be studied [68].

Additionally, miR-150 may regulate fibroblast autophagy via ER stress and function in IBD intestinal fibrosis. Studies have shown that IRE1a has a splicing effect on XBP1. The IRE1*α*-XBP1 axis leads to the expansion of the ER, enhancing its ability to secrete extracellular matrix proteins and activate myofibroblasts. An increase in *α*SMA expression is a characteristic of fibroblast transformation into myofibroblasts. miR-150 inhibits expression of the transcription factor c-Myb (the primary target of miR-150, which can increase expression of *α*SMA), thereby reducing expression of *α*SMA to exert an antifibrosis effect. Although IRE1*α* can directly regulate degradation of miR-150, the loss of IRE1*α* activity may lead to an increase in miR-150 levels [69]. Because the absence of *XBP1* enhances activation of IRE1 during endoplasmic reticulum stress [59], expression of miR-150 is speculated to be reduced, and any antifibrosis effect is expected to be weakened. miR-150 is expressed in the serum of IBD (UC) tissues [4] and may thus regulate fibroblast autophagy via ER stress and play a role in IBD intestinal fibrosis (Figure 2).

IRE1 interacts with TRAF2 to activate JNK signaling, and JNK directly induces formation of autophagosomes via LC3, leading to enhanced autophagy. The IRE1*α*-XBP1 axis leads to the expansion of the endoplasmic reticulum, enhancing its ability to secrete extracellular matrix proteins and activate myofibroblasts. XBP1 deficiency in endoplasmic reticulum stress leads to enhanced IRE1 activation, decreased miR-150 expression, and reduced c-Myb inhibition, leading to greater expression of *α*SMA, expansion of the endoplasmic reticulum, enhancement of extracellular matrix protein secretion capacity, and activation of fibroblast transformation into myofibroblasts, ultimately leading to fibrosis.

## 4. Signaling Pathway

### 4.1. NF-*κ*B, MAPK (JNK), and FOXO3a

NF-*κ*B activates a variety of signaling molecules, including Toll-like receptors and cytokine receptors, which play an important role in inflammation, immune responses, cell proliferation, cell differentiation, and apoptosis [70]. Autophagy inhibits NF-*κ*B activation, and NF-*κ*B suppresses autophagy [34]. Autophagy attenuates NF-*κ*B in inflammation, whereas inhibition of autophagy activates NF-*κ*B by blocking the formation of autophagosomes and promoting autophagosome accumulation. Activation of NF-*κ*B also induces the upregulation of p62 and JNK to form a positive feedback loop that inhibits autophagy [71, 72]. c-Jun N-terminal protein kinase (JNK) is a serine/threonine kinase and mitogen-activated protein kinase (MAPK) in mammals that is a core component of the autophagic signaling pathway. Phosphorylated JNK promotes autophagy through upstream Bcl2 family protein kinases [73]. *FoxO3a* enhances autophagy in response to environmental stress and activates autophagy by binding directly to the promoters of autophagy-related genes such as *LC3*, *Beclin1*, and *Atg12*. *FoxO3a* can also be phosphorylated and inactivated by PI3K/Akt. Akt phosphorylation reduces *FoxO3a* transcriptional activation by inhibiting *FoxO3a* translocation to the nucleus. Autophagy might be induced by the Akt/FoxO3a pathway to protect cells in the early stage of environmental stress. Autophagy is decreased in the later stage, but FoxO3a expression continues to increase, subsequently upregulating p-Akt expression [74]. Additionally, mTOR enhances the NF-*κ*B signaling pathway by downregulating expression of *FOXO3*, suggesting an ability to inhibit autophagy [75].

Studies have shown that miR-146b overexpression activates and upregulates the NF-*κ*B pathway, thereby inhibiting autophagy, improving intestinal epithelial function, reducing intestinal inflammation in dextran sulfate sodium- (DSS-) induced colitis mice, and increasing the survival rate of fatal colitis [76]. Substance P (SP) also increases NF-*κ*B pathway activation in colonic epithelial cells [77, 78] by regulating expression of miR-221-5p via the MAPK and NF-*κ*B pathways, and miR-221-5p negatively regulates expression of proinflammatory cytokines in colonic epithelial cells by responding to SP. The increased expression of miR-221-5p in human and mouse colitis tissues, which elevates SP expression, suggests a role in IBD autophagy [79]. Moreover, because they are upregulated in IBD patients and in mouse inflammatory bowel tissues, miR-132 and miR-223 have been reported to be important mediators in IBD pathogenesis. They can inhibit expression of I*κ*B*α* by negatively regulating *FOXO3a*, leading to the enhancement of NF-*κ*B signaling and increasing the production of proinflammatory cytokines [80]. In another study [81], JNK signaling was shown to be upstream of miR-223, and activation of JNK induced miR-223 expression. Therefore, we speculate that JNK signaling is activated in IBD and that it upregulates miR-223 expression, leading to the enhancement of NF-*κ*B signaling and downregulation of *FOXO3a*. This process plays a role in inhibiting autophagy. In addition, miR-21 is overexpressed in intestinal inflammation and tissue injury, which can suppress autophagy (see Section 3.2). Upregulation of miR-21 has been found in ovarian cancer cells in association with activation of the JNK-1/c-Jun pathway [82]. Nonetheless, it remains unclear whether these mechanisms regulate (inhibit) autophagy in IBD.

### 4.2. mTOR, Akt, PI3K, and PTEN

Mammalian target of rapamycin (mTOR) is a serine/threonine kinase. Inhibition of mTORC1-induced autophagy using genetic or pharmacological methods was first achieved in yeast studies [83]. In mammals, mTORC1 inhibits autophagy initiation through phosphorylation [84, 85]. Akt is an intracellular serine/threonine kinase that is downstream of phosphatidylinositol-3 kinase (PI3K). Phosphorylated Akt (p-Akt) activates mTOR, as reflected by an increase in p-mTOR expression, leading to autophagy inhibition [86]. Related studies have shown that miR-155 can increase Akt activation by decreasing expression of SHIP-1, resulting in enhanced mTORC1 expression, and inhibition of miR-155 can reduce the inflammatory reaction in experimental colitis. The PI3K-AKT signaling pathway was strongly activated by sustained overexpression of miR-155 in DHL16 cells [75, 87–89]. However, whether miR-155 plays a role in autophagy in IBD remains to be studied. Knockdown of mouse miR-21 reduces the effect of inflammation on tissue damage and increases survival rates in DSS-induced lethal colitis [90]. miR-21 has also been shown to specifically inhibit autophagy in a variety of diseases. For example, overexpression of Rab11a increases expression of LC3II and beclin-1 in renal ischemia. miR-21 directly inhibits Rab11a expression, thereby inhibiting autophagy, and participates in the Akt/PTEN pathway to inhibit autophagy in primary liver cancer [90–94]. In contrast, some studies have shown that miR-21 inhibits p-Akt and deactivates mTOR to induce autophagy [95, 96]. *PTEN*, a potential target gene of miR-21, acts as a lipid phosphatase that antagonizes PI3K signaling. miR-21 inhibition increases *PTEN* protein levels and suppresses AKT phosphorylation [95, 97]. More research is needed to determine whether miR-21 inhibits or induces autophagy in IBD via these pathways. One study has also demonstrated that miR-106b and miR-93 regulate cell progression by suppressing *PTEN* and enhancing activity of the PI3K/Akt pathway. Regardless, it remains to be verified whether these miRNAs play the same role in IBD [98].

## 5. Conclusion

The important role of miRNA in IBD pathophysiology has been clarified in recent years with increasing molecular evidence on IBD occurrence and development. Current studies on miRNA-related mechanisms in IBD mainly focus on three components: immune homeostasis disorder, intestinal epithelial barrier dysregulation, and autophagy regulation [16]. However, few studies have attempted to identify which miRNA may be more linked to autophagy in IBD, and the functions of miRNAs in the autophagy pathway in IBD have only been addressed with cellular experiments but few in vivo experiments. In addition, the mechanisms of the *NOD2* and NF-*κ*B pathways appear to have garnered the most attention. It is likely that other signal pathways will be linked to IBD, including the classic autophagy pathway LKB1/AMPK-PI3K/AKT and beclin-1/bcl-2. Therefore, more studies must be performed in the future to expand these fields. Additionally, although many studies have demonstrated that miRNAs regulate related proteins in the autophagy pathway, only a few studies have investigated the effect of miRNA on autophagy flux. As autophagy is a dynamic biological process, it would be interesting to elucidate the role of miRNAs in autophagic flux.

Study of the molecular expression mechanisms of miRNAs in IBD may lead to IBD molecular targeted therapy. Given the function of miRNAs in regulating autophagy in IBD pathogenesis, exploring the potential of miRNA-autophagy-based therapeutics is also needed. As it has been identified as a new diagnostic/prognostic tool, miR-21 may serve as a therapeutic target in the future. Nonetheless, before application of this basic research to the clinic can occur, many technical issues need to be overcome. For example, a single miRNA can interact with multiple genes or tissues in vivo, and it may be involved in multiple signaling pathways. Using inhibitors may lead to suboptimal side effects by affecting many pathways in different type of cells. Therefore, identification of IBD-associated miRNA autophagic networks and exploration of specific miRNAs as targets for the treatment of IBD is very important. There are two completed phase I trials (NCT00688012 for a single ascending dose and NCT00979927 for multiple ascending doses; drug: SPC3649) funded by Santaris Pharma related to a locked nucleic acid- (LNA-) modified oligonucleotide that specifically inhibits endogenous miR-122, resulting in prolonged dose-dependent reductions in hepatitis C virus (HCV) RNA levels without evidence of viral resistance [25, 100].

In summary, both miRNAs and autophagy play an important role in IBD. The effect of miRNA on autophagy is a promising field of study for exploring IBD treatment. A deeper understanding of the complex dialog between miRNAs and autophagy and their exact roles in IBD may lead to a great success in the future.

## Figures and Tables

**Table 1:** Summary of miRNAs that regulate autophagy in IBD.

MicroRNA	Samples	Diseases		Target	Effects on autophagy	Potential mechanism	Years and references
		CD	UC				
miR-142-3p^**※**^	HCT116	**+**	**−**	ATG16L1, NOD2	Inhibit	Decreases ATG16L1 mRNA and protein levels; modulates NOD2-dependent autophagy; inhibits IL8 mRNA expression.	2014 [30] 2018 [40]
miR-320	HT-29	**+**	**+**	NOD2, NF-*κ*B	Inhibit	Downregulates NOD2 expression.	2016 [31]
miR-192^**※**^	HCT116	**+**	**−**	NOD2, NF-*κ*B	Inhibit	Downregulates NOD2 expression; suppresses NF-*κ*B activity.	2014 [33]
miR-122	HT-29	**+**	**−**	NOD2, NF-*κ*B	Inhibit	Suppresses NOD2; increases NF-*κ*B activity.	2017 [35] 2013 [36]
miR-93; miR-106B	Human colon tissues, HCT116	**+**	**−**	ATG16L1 PTEN	Inhibit	Targets ATG16L1 messenger RNA; reduces levels of ATG16L1; prevents autophagy-dependent eradication of intracellular bacteria; suppresses PTEN by enhancing activity of the PI3K/Akt pathway.	2014 [41] 2017 [98]
miR-30C; miR-130a	Human intestinal epithelial T84 cells/mice enterocytes	**+**	**−**	ATG5, ATG16L1 mTOR	Inhibit	Reduced levels of ATG5 and ATG16L1; miR-130a increases levels of p-mTOR; impairs LC3-II accumulation.	2014 [44] 2017 [43]
miR-346	Human intestinal epithelial cells	**+**	**+**	Vitamin D receptor (VDR) GSK3B	Upregulate	Downregulates VDR, leading to upregulation of ATG16L1; activates autophagy by interrupting the association between BCL2 and BECN1 in a GSK3B-dependent manner.	2014 [46] 2018 [68]
miR-20a	Human colonic mucosal tissues	**+**	**−**	BECN1, ATG16L1, SQSTM1	Inhibit	Downregulates BECN1, ATG16L1, and SQSTM1.	2017 [47, 48]
MiR-196^**※**^	Human epithelial cells	**+**	**−**	LC3-II, IRGM	Inhibit	Inhibits accumulation of LC3-II.	2011 [53]
miR-665	Human/mice colonic mucosal tissues.	**+**	**+**	XBP1, ORMDL3	Induce	Represses XBP1 and ORMDL3 expression.	2017 [65]
miR-150	Intestinal tissue serum	**−**	**+**	c-Myb	Unclear	Inhibits c-Myb, leading to decreased *α*SMA; exhibits antifibrotic effects.	2016 [69]
miR-146b	Mice colonic tissues.	**−**	**+**	siah2 FOXO3	Inhibit	Decreases expression of siah2; activates the NF-*κ*B pathway. Decreases siah2 and FOXO3 expression.	2013 [76] 2016 [99]
miR-221-5p	Human colonic epithelial cells	**+**	**+**	Substance P	Inhibit	SP regulates miR-221-5p expression through the MAPK and NF-*κ*B pathways; miR-221-5p negatively regulates expression of proinflammatory cytokines in colonic epithelial cells in response to SP.	2014 [79]
miR-132; miR-223	Human/mice colonic tissues	**+**	**+**	FOXO3a, JNK	Inhibit	Suppresses the level of I*κ*B*α* through downregulation of FOXO3a, leading to enhanced NF-*κ*B signaling; JNK signaling induces miR-223.	2016 [80] 2015 [81]
miR-155^**※**^	Mice colonic tissues	**−**	**+**	SHIP-1, FOXO3a PI3K-Akt pathway	Inhibit	Increases Akt activation by decreasing SHIP-1 expression, leading to upregulation of mTOR; downregulates FOXO3a, leading to enhanced NF-*κ*B signaling; activates the PI3K-Akt pathway.	2017 [87] 2015 [75] 2012 [89]
miR-21^**※※**^	Mice colonic tissues.	**−**	**+**	Akt, m-TOR, JNK, PTEN	Inhibit/induce	Unclear/decreases phosphorylated AKT and deactivates the mTOR NK-1/c-Jun pathway; promotes miR-21 upregulation. miR-21 inhibition enhances PTEN protein levels and inhibits AKT phosphorylation. Inhibits autophagy via the PTEN/Akt/mTOR pathway.	2013 [90, 96] 2015 [95] 2014 [82] 2018 [97]

※ refers to more closely related to IBD; ※※ refers to the most studied in IBD [8, 16, 25].
